# Identification, description and appraisal of generic PROMs for primary care: a systematic review

**DOI:** 10.1186/s12875-018-0722-9

**Published:** 2018-03-15

**Authors:** Mairead Murphy, Sandra Hollinghurst, Chris Salisbury

**Affiliations:** Population Health Sciences, Bristol Medical School, University of Bristol, Canynge Hall, 39 Whatley Road, Bristol, BS8 2PS UK

**Keywords:** Systematic review, Patient-reported outcomes, Primary care, Questionnaires, Generic PROMs, Transitional PROMs

## Abstract

**Background:**

Patients attend primary care with many types of problems and to achieve a range of possible outcomes. There is currently a lack of patient-reported outcome measures (PROMs) designed to capture these diverse outcomes. The objective of this systematic review was to identify, describe and appraise generic PROMs suitable for measuring outcomes from primary care.

**Methods:**

We carried out a systematic Medline search, supplemented by other online and hand-searches. All potentially relevant PROMs were itemised in a long-list. Each PROM in the long-list which met inclusion criteria was included in a short-list. Short-listed PROMs were then described in terms of their measurement properties and construct, based on a previously published description of primary care outcome as three constructs: health status, health empowerment and health perceptions. PROMs were appraised in terms of extent of psychometric testing (extensive, moderate, low) and level of responsiveness (high, medium, low, unknown).

**Results:**

More than 5000 abstracts were identified and screened to identify PROMs potentially suitable for measuring outcomes from primary care. 321 PROMs were long-listed, and twenty PROMs were catalogued in detail. There were five PROMs which measured change directly, without need for a baseline. Although these had less strong psychometric properties, they may be more responsive to change than PROMs which capture status at a point in time. No instruments provided coverage of all three constructs. Of the health status questionnaires, the most extensively tested was the SF-36. Of the health empowerment instruments, the PEI, PAM and heiQ provided the best combination of responsiveness and psychometric testing. The health perceptions instruments were all less responsive to change, and may measure a form of health perception which is difficult to shift in primary care.

**Conclusions:**

This systematic review is the first of its kind to identify papers describing the development and validation of generic PROMs suitable for measuring outcomes from primary care. It identified that: 1) to date, there is no instrument which comprehensively covers the outcomes commonly sought in primary care, and 2) there are different benefits both to PROMs which measure status at a point in time, and PROMs which measure change directly.

**Electronic supplementary material:**

The online version of this article (10.1186/s12875-018-0722-9) contains supplementary material, which is available to authorized users.

## Background

### Introduction

Patient-reported outcome measures (PROMs) are self-report questionnaires designed to capture information on patients’ health. An ‘outcome’ is change in health status, knowledge or behaviour which is attributable to preceding healthcare [1], and PROMs provide important evidence about this change as it is experienced by the patient [2]. There are thousands of PROMs in existence, with new PROMs being developed every day [3]. Experts in the field have called for harmonisation in this area [4], including research into existing instruments before development of new ones [5].

PROMs were originally developed to aid in evaluating and comparing the effectiveness of healthcare interventions [3]. By comparing patients’ PROM scores before and after an intervention, the outcome of the intervention can be assessed. Numerous primary care interventions have been developed in recent years to meet changing population and service needs (including an aging population and increasing numbers of people with multi-morbidity [6, 7]). While there are a number of disease and problem-specific PROMs which can be used in primary care, many primary care interventions are targeted at people with a range of conditions or problems. Examples include electronic consultations [8], health coaching and behavioural change therapies [9, 10], and new approaches to address the needs of frequent attenders in general practice [11, 12]. Assessing the effectiveness of such interventions from a patient perspective requires a *generic* PROM, which can be administered across a population, regardless of presenting problem. Such a PROM should cover multi-layered outcomes encompassing aspects of enablement, resilience, symptoms and function, and health perceptions.

We conjectured that there was no such suitable PROM for primary care and undertook to investigate this through a review of the literature. We identified existing structured reviews of PROMs on related topics: for example PROMs for mental health [13], empowerment [14, 15], integrative medicine [16], patient experience [17] patient safety in primary care [18] and generic health status in older people [19]. However, an initial search of the literature found there was no structured review for generic PROMs specific to the measurement of outcome across all primary care patients.

We firstly carried out a qualitative study to delineate the domains which should be captured by a Primary Care PROM [20]. We then conducted a systematic review of PROMs suitable for primary care, which captured these domains.

### Prior qualitative study

In our prior Qualitative study, we identified and categorised inter-related outcomes into ten groups occupying three domains:

1. **Health Status**: This involves both symptoms and medication side-effects and the impact of symptoms on patients’ lives.
2. **Health Empowerment**: These are the internal and external resources which enable patients to improve their health. The internal resources include an understanding of health conditions, and an ability to self-care, stay healthy and follow a clinician-patient agreed plan. The external resources include patients’ confidence in seeking healthcare, and ability to access suitable health-related supports. Although these external aspects are closely related to the patient experience of the consultation, they are the enduring impacts of the consultation that have a direct influence on patient’s overall health status and are qualitatively different from measures of patient experience [20].
3. **Health Perceptions**: This involves health concerns and satisfaction, and confidence in their health for the future.

This study reports on a systematic review of PROMs suitable for use in primary care to measure these outcomes.

## Methods

### Search strategy

We designed a customised search strategy for Medline Ovid SP, following PRISMA guidelines where appropriate [21]. This was peer-reviewed by a University of Bristol librarian and included indexed papers from 1950 to 8th March 2014. The PICO framework normally used in systematic reviews (population, intervention, comparator and outcomes) [22] was adapted in order to identify primary care PROMs; the framework used was: Population, Aim, Subject and Construct (PASC). Four filters were developed using these PASC categories combined with an AND operator. The population filter was designed to retrieve papers relevant to primary care; the aim and subject filters combined were designed to retrieve papers describing development and validation of PROMs; and the construct filter was aimed at the domains of interest (health status, health empowerment, and health perceptions). Search terms for each category were developed through an iterative process of adjusting filters and performing test searches. A full description of the four filters is shown in Additional file 1.

We recognised that limiting the search to the Medline database meant some relevant papers may have been missed. We therefore followed-up all PROMs referred to in screened abstracts, contacted eighteen experts in the field, hand-searched three compilations of PROMs (McDowell [23], Bowling [24], and PROQOLID [25]) and screened all abstracts on the Oxford University PROMs group database using the keywords “individualised”, “generic”, “utility” or “primary care”. (This a database of papers relating to patient-reported outcome measures, which contains references to more than 14,000 papers, last updated in 2005) [26]. A backwards reference search was carried out on all twenty original papers included in the final review, and a forward reference search for sixteen of the twenty original papers. (The four exceptions were those which had been cited more than 600 times).

### Selection of PROMs

All abstracts identified in the electronic searches were screened. During this process, any PROMs named in the abstract were listed, apart from those which did not meet the inclusion criteria.

For each of the PROMs in the long list, a copy of the instrument was obtained from either a PROMs compilation [23, 24, 26, 27] or the initial development paper. Selection was based on inclusion and exclusion criteria on the *PROMs*, as opposed to the *papers* (see Table 1). To ensure decisions were made consistently, reasons for exclusion were documented against each PROM. (See Additional file 2).

**Table 1:** Inclusion and exclusion criteria for long-listed PROMs

	Inclusion criteria	Exclusion criteria
Construct	1. PROMs which provide wide coverage of at least one domain as specified from the Qualitative Study.	1. Construct: PROMs excluded on the basis of construct may be: a) unrelated constructs – e.g. adjustment to illness, b) related, but covering only a single sub-domain (e.g. pain, adherence), c) related, but mostly comprising of constructs which were not identified in the Qualitative Study (e.g. quality of life, personality traits).
Population	2. PROMs which have been used in primary care, or for patients with chronic conditions / minor illness. 3. PROMs suitable for use in adults, across both sexes, any disease and any presenting health problems.	2. Disease Specific (e.g. asthma). 3. Population-specific (e.g. the elderly, the functionally limited, children). 4. Intervention-specific (e.g. occupational health, social care).
Administration	4. PROMs which can be self-administered, and are self-reported.	5. Not patient-reported (e.g. clinician-reported, proxy-reported and strictly interview administered).
Other Criteria	5. Instruments in English which can be completed in ten minutes or less.	6. Not available in English. 7. Too long (longer than 50 items).

**Fig. 1:**
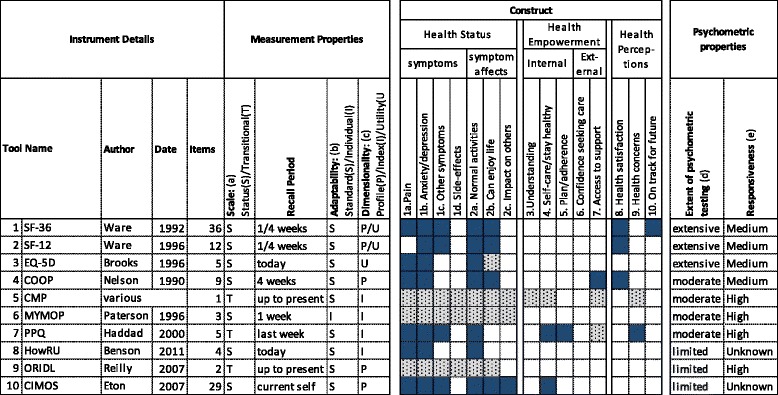
Health Status Instruments Reviewed. 1 (SF-36): MOS Short Form 36v2 [34, 35]; 2 (SF-12): MOS Short Form 12 [36]; 3 (EQ-5D): EuroQol 5D [39]; 4 (COOP): Dartmouth COOP Charts [37]; 5 (CMP) Change in Main Problem [45–47] 6 (MYMOP): Measure Yourself Medical Outcomes Profile v2 [43]; 7 (PPQ): Patient Perception of Quality [32] 8 (HowRU) HowRU [50]; 9 (ORIDL) Outcomes Related to Impact on Daily Living [33]; 10 (CIMOS) Complementary and Integrative Medical Outcomes Scale [52]. Scale (a) S = Status (capturing status at a point in time). T = Transitional (capturing change over a period of time). Adaptability (b) S = Standardised (standard list of items) I = Individualised (respondents can select, identify or weight items). Dimensionality (c) P = Profile of scores. I = Index (single score). U = Utility (single preference-based score which can generate a QALY). Extent of psychometric testing (d) Extensive (Widespread validation in different populations/countries and/or > 1000 citations). Moderate (Independent validation and/or > 100 citations). Low (Validation by original authors and/or < 100 citations). Responsiveness (e) Unknown (responsiveness not known or tested). Low (responsiveness shown in at least one study). Moderate (Repeated evidence for responsiveness, including in primary care). High (responsiveness shown in primary care studies where other leading PROMs are not responsive)

**Fig. 2:**
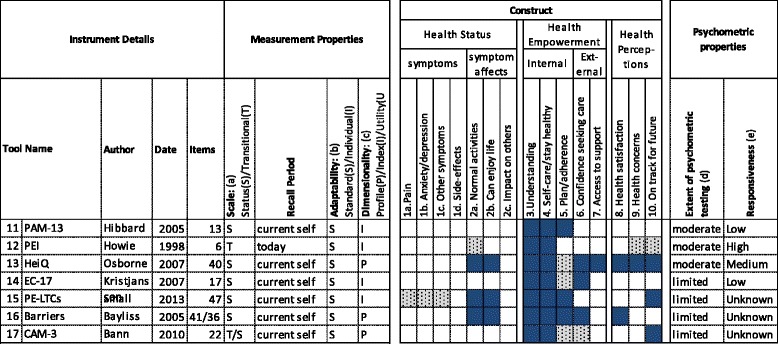
Health Empowerment Instruments Reviewed11 (PAM-13): Patient Activation Measure [55]; 12 (PEI): Patient Enablement Instrument [58]; 13 (heiQ): Health Education Impact Questionnaire [56]; 14 (EC-17): Effective Consumer Scale [59]; 15 (PE-LTCs): Patient Empowerment in Long-Term Conditions [61]; 16 (Barriers): Barriers to Self-Care in Multiple Long-Term Conditions [62]; 17 (CAM-3) Three scales for Complementary and Alternative Medicine [63]. Scale (a) S = Status (capturing status at a point in time). T = Transitional (capturing change over a period of time). Adaptability (b) S = Standardised (standard list of items) I = Individualised (respondents can select, identify or weight items). Dimensionality (c) P = Profile of scores. I = Index (single score). U = Utility (single preference-based score which can generate a QALY). Extent of psychometric testing (d) Extensive (Widespread validation in different populations/countries and/or > 1000 citations). Moderate (Independent validation and/or > 100 citations). Low (Validation by original authors and/or < 100 citations). Responsiveness (e) Unknown (responsiveness not known or tested). Low (responsiveness shown in at least one study). Moderate (Repeated evidence for responsiveness, including in primary care). High (responsiveness shown in primary care studies where other leading PROMs are not responsive)

**Fig. 3:**
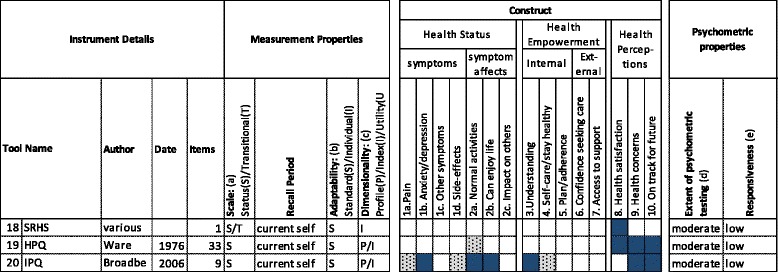
Health Perceptions Instruments Reviewed. 18 (SRHS): Single item indicator of self-rated health status [27]; 19 (HPQ): RAND Health Perceptions Questionnaire [73]; 20 (IPQ): Illness Perceptions Questionnaire [74]. Scale (a) S = Status (capturing status at a point in time). T = Transitional (capturing change over a period of time). Adaptability (b) S = Standardised (standard list of items) I = Individualised (respondents can select, identify or weight items). Dimensionality (c) P = Profile of scores. I = Index (single score). U = Utility (single preference-based score which can generate a QALY). Extent of psychometric testing (d) Extensive (Widespread validation in different populations/countries and/or > 1000 citations). Moderate (Independent validation and/or > 100 citations). Low (Validation by original authors and/or < 100 citations). Responsiveness (e) Unknown (responsiveness not known or tested). Low (responsiveness shown in at least one study). Moderate (Repeated evidence for responsiveness, including in primary care). High (responsiveness shown in primary care studies where other leading PROMs are not responsive)

Abstract screening and data extraction was done by a single reviewer (MM). The other two reviewers (CS/SH) independently checked the extracted data (shown in Figs. 1, 2 and 3), and reviewed the long-list of PROMs excluded from the review. (Additional file 2).

### Data extraction

The selected PROMs were described in a tabular format (see Figs. 1 to 3). Data was extracted on their measurement properties, construct and psychometric properties.

The measurement properties extracted were adapted from an existing PROM classification framework [25], and included the number of items, the nature of the scale, the recall period, the level of PROM adaptability and the dimensionality.

The construct categories extracted were based on the prior qualitative study [20]. Where a construct was explicitly covered, this was block-highlighted in the tabular description. Where it was implicit (for example, an individualised questionnaire which asks about symptoms covers pain, but not explicitly) it was shaded.

The review of psychometric properties was limited to the *extent* of psychometric testing, and the *level* of responsiveness. PROMs were categorised as having an extensive, moderate or low extent of psychometric testing, depending on the number of validation studies published, whether the authorship of these papers extended beyond the original authors, and on number of times the original development paper has been cited. (see additional file 3 for details). With the exception of responsiveness, we chose not to provide categorical ratings for individual psychometric properties (for example, validity, reliability, interpretability) because these are actually properties of PROMs *as administered in a particular population*, not of property of a PROM in and of itself [28]. Although some structured reviews of PROMs have provided such categorical ratings[16], leading textbook compilations appraise these properties descriptively [23, 27] and we also took this approach. We made an exception for responsiveness, because the current study was based on the hypothesis that there is no suitably responsive PROM for testing interventions in primary care, so it was necessary to test this hypothesis. We categorised responsiveness as: unknown (responsiveness not known or tested); low (responsiveness shown in at least one study); medium (repeated evidence for responsiveness, including in primary care); high (responsiveness shown in primary care studies where other leading PROMs are not responsive).

PROM compilations [23, 24, 26, 27] were reviewed to extract psychometric information relating to the most common PROMs (e.g. the SF-36). Where the PROM was not listed in a complication, a forward reference search on the original PROM development paper was used.

A fuller description of the data extraction sheet is shown in Additional file 3.

## Results

### Search and selection of PROMs

**Fig. 4:**
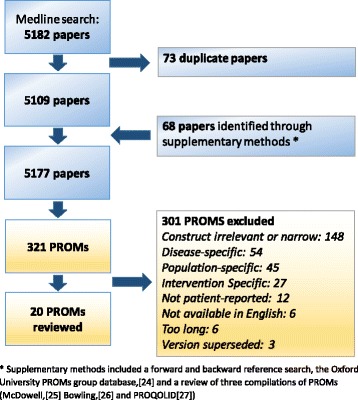
Papers and PROMs identified through the systematic review

Figure 4 shows the number of papers screened and PROMs identified. Many PROMs were excluded at short-listing stage because of the construct or the population. For example, the Sickness Impact Profile was excluded because it is most suitable for very ill populations [27]. Although several preference-based instruments were long-listed, only the *EQ-5D* and *SF-36*/*SF-12* were included in the final review. ICECAP [29] and AQoL-4D [30] were excluded because of their focus on general quality of life, which was not part of the construct under consideration. The Health Utilities Index was excluded because of its deliberately narrow focus on specific aspects of function [27].

The twenty selected PROMs are described in the following three sections by domain: Health Status, Health Empowerment and Health Perceptions. Where a PROM covered more than one domain, it is presented under the domain with which it has most overlap. A referenced list of the twenty PROMs can be found in Additional file 4.

### Health status PROMs

#### Construct and measurement properties

Ten instruments which measure some form of health status were included. As shown in Fig. 1, nine of the ten instruments contain a standard list of questions. One of the instruments (*MYMOP*) is individualised, such that patients define the outcomes of interest in their own words [31]. Three of the PROMs contain transitional items: the *CMP*, *PPQ* [32] and *ORIDL* [33].

Five of the instruments result in a profile of scores. The most widely used and well-validated of these is the SF-36, which measures physical and emotional function [34, 35]. The second profile PROM (the *SF-12)* was designed as a short version of the *SF-36* [36] and was validated based on assessing how well the twelve-item scale scores predicted the 36-item scale scores [36]. The third most commonly cited profile instrument listed is the Dartmouth *COOP charts*. These were designed as a rapid way to assess functional health routinely in clinical practice [37] and consist of a set of nine charts, each with a title, a question, and a five-point pictorial response scale. The fourth profile reviewed is the transitional *ORIDL*. This consists of two scores designed to be comparable across different people and diseases [33]. The last profile is the Complementary and Integrative Medicine Outcome Scales (*CIMOS*) [38]. This instrument was developed to measure the outcomes typically experienced by people receiving complementary and alternative medicine. Four of the instruments generate index (but not utility) scores. These are the *CMP*, *howRU*, *MYMOP* and *PPQ*. Three instruments generate utility scores: the, SF-36, SF-12 and the EQ-5D [39–41] (which is recommended by NICE as the tool of choice for economic evaluation [42]).

#### Psychometric properties

The *SF-36, SF-12* and the *EQ-5D* have been extensively used and tested, with the original papers describing each cited approximately 21,000, 9000 and 3000 times respectively. The *COOP Charts*, the *CMP* and *MYMOP* have undergone moderate levels of psychometric testing. The original *PPQ* French version has also had moderate testing, but testing of the English version has been more limited. The remaining three instruments have had limited testing.

The most responsive to change are the individualised instrument (*MYMOP*), and the three transitional instruments (*PPQ*, *CMP* and *ORIDL*). *MYMOP* shows change when the *SF-36* does not [31, 43, 44]. Various formats of the *CMP* have been used in primary care trials [45–47]. However, as a single-item, this has lower reliability than multi-item instruments [28]. *ORIDL* is also transitional. Although limited testing has been done of *ORIDL*, in the initial validation study, it showed good correlation with *MYMOP* and *PEI* [33]. One study showed that *ORIDL* continued to show change on repeated follow-up when *MYMOP* did not [48].

The *SF-36*, *SF-12, EQ-5D* and the *COOP charts* all have a medium level of responsiveness. However, there is considerable variability within this. The most responsive of these instruments is the *SF-36* profile scores [27]. The *COOP charts* show good reliability and validity in primary care populations. Unsurprisingly, given that each profile score is based on a single item, they are less reliable than the *SF-36*, have a ceiling effect, and are less responsive to change over time [27]. In terms of preference-based values, testing has showed that the recent *SF-12* value set is just as responsive to change and generates similar estimates to the *SF-36* preference-based index [49]. A study of patients with depression also showed the *SF-12* utility scores to be more responsive than the *EQ-5D-3 L* which suffers from ceiling effects in general [27].

*HowRU* [50], has not been tested for responsiveness. However, it has shown less of a ceiling effect than *EQ-5D-3 L*, despite being even shorter [51]. This may be because of wording e.g. “low or worried” (howRU) rather than “anxious or depressed” (EQ-5D). Responsiveness is also unknown in *CIMOS*, which has undergone very little psychometric testing, demonstrating acceptable levels of reliability and validity in only one study [52].

#### Summary

The *SF-36* is, by far, the most validated of the health status instruments and the most responsive of the moderately and extensively tested PROMs. *MYMOP* and *ORIDL* both represent good attempts to increase responsiveness, but the *ORIDL* detailed nine-point scale has not been widely validated, and, although it has been used in trials [47, 53], MYMOP is not recommended for self-completion, which is necessary for routine or trial use [31]. *EQ-5D* and *SF-12* have the benefits of brevity, and the ability to generate a preference-based score. *HowRU* shows that it is possible to have a valid instrument that is very short. Haddad’s transitional scale provides another good option for increasing responsiveness while maintaining a standard list of items.

### Health empowerment

#### Construct and measurement properties

Figure 2 shows the seven instruments which cover both internal and external aspects of the Health Empowerment construct.

The constructs measured by these seven instruments all include internal aspects of empowerment, with explicit items on understanding of health problems, and the ability to self-care, or stay healthy. External aspects of empowerment are less extensively covered, perhaps because these are traditionally seen as measures of patient experience, not outcome [54], and because we did not include measures which exclusively capture patient experience in our review. None of the instruments directly address symptoms. The three most widely used are *PAM-13*, *PEI* and the *heiQ*. *PAM-13* is based on a single construct of activation: which is being engaged in managing one’s own health. Patients are measured on a four-stage Guttman scale of activation: from belief that an active role is important, to taking action and staying the course under stress [55]. The *heiQ* was developed to assess the impact of patient education programs across a broad range of chronic conditions [56]. It has a wider construct than the *PAM-13* and contains eight independent dimensions: positive and active engagement in life, health directed behaviour; constructive attitudes and approaches; self-monitoring and insight; health service navigation; social integration and support and emotional wellbeing [56, 57]. These domains overlap with both internal and external empowerment, and also with the other two domains. For example, “positive and active engagement in life” overlaps with Health Status. The instrument also includes aspects of Health Perceptions, including “satisfaction with health”, and “health concerns”. *PEI* was developed specifically for primary care, and asks the patient to retrospectively rate change in enablement, resulting from a single consultation. As well as understanding and self-care, it addresses concerns, and indirectly addresses the impact of symptoms (through questions on coping with illness, and coping with life) [58].

The four remaining instruments are less widely used. *EC-17* was developed to measure the skills and attributes of an effective consumer, for use in self-management interventions [59, 60]. *PE-LTCs* was developed to measure empowerment in long-term conditions [61]. The *Barriers* instrument does not purport to assess empowerment, rather barriers to self-management in long-term conditions [62]. However, the construct of “barriers” is related to empowerment, in that reducing barriers increases empowerment. *CAM-3* measures the quality of the therapeutic relationship as: 1) patient-centred care, 2) perceived provider support 3) empowerment. While this is described as an experience measure, it focuses on the consequences of a positive experience, for example, trust in the therapist and belief that the root causes are being identified and treated. In measuring patient-centred care and perceived provider support as well as empowerment, it includes some external aspects of empowerment in addition to internal [63].

All seven instruments contain a standard list of questions, asking about today, or a person’s perception of their current self. Two of the instruments are, at least partly, transitional. The status instruments are based on a list of belief statements with a Likert (bipolar) response scale (e.g. strongly disagree to strongly agree) apart from the *EC-17*, which has behavioural statements with an adjectival scale (never to always). Four of the instruments provide a single index score, and the remaining three give a profile of scores. All instruments are scored using a summative method, apart from *PAM-13*, which uses a Rasch scoring algorithm [55].

#### Psychometric properties

The first three of the instruments (*PAM-13*, *PEI* and *heiQ*) have undergone moderate levels of testing. *PEI* has been used widely in UK general practice and has shown acceptable psychometric properties. As a transitional questionnaire, it measures change directly, and is thus responsive.

The properties of the *heiQ* were investigated using item response theory and structural equation modelling. It has demonstrated good construct validity, including, most recently, testing for measurement invariance [64]. Some of the *heiQ* sub-scores have shown responsiveness to change in randomised controlled trials [65–67].

*PAM-13* has strong psychometric properties, and association with a number of other health outcomes [68]. Recent studies in the US found patient activation was influenced by community interventions [69, 70], suggesting it may be appropriate as an outcome measure in primary care.

The *EQ-17* has shown some preliminary evidence for responsiveness to change in arthritis patients [60, 71], although psychometric testing has been more limited. However, the authors of the instrument acknowledge that, while some skills of an effective consumer can be learned, others are a “part of personality” and not amenable to change [59]. (pg. 1932) When compared directly to *PAM-13* it was less responsive (standardised response mean 0.25/ 0.41).

#### Summary

All the health empowerment instruments reviewed could, in theory, be used to measure empowerment outcomes in primary care. However, as all except *PEI* and *CAM-3* were developed with long-term conditions in mind, they are less suitable for people without long-term conditions. This is most problematic with *EC-17*, *PE-LTCs* and the *Barriers* questionnaire, which all refer to a “disease”. The first three instruments (*PAM-13*, *PEI* and *heiQ*), which are the most robust and responsive to change, make minimal reference to “your illness” or else refer to “health problems” in general. Of these three, only the *PEI* was developed specifically for primary care. The main weakness of this is that it only works at a single consultation level, through the words “as a result of your consultation today”. A format of the *PEI* which asks patients to rate a longer episode of care has been tested for acupuncture. This adjusted the wording to “as a result of visiting the acupuncturist over the last few weeks or months.” However, patients had difficulty attributing change directly to the intervention [72]. *PAM-13*, is more robust, but the construct is relatively narrow: its emphasis is on the internal, and it contains elements about control and responsibility which are not present in the construct described in Chapter 3. The *heiQ* has the widest construct. The main weakness of the *heiQ* for use in primary care is its length, and the fact that it does not explicitly address symptoms, which, for some patients, may be the primary reason for attendance.

### Health perceptions

#### Construct and measurement properties

Three instruments which predominantly cover a Health Perceptions construct were identified (Fig. 3). The single *self-rated health status* item is based on empirical evidence that people possess insights into their own health, and that this can be captured through a single rating of how they perceive their health at a point in time [27]. *Self-rated health status* items ask for a general impression of health, rather than symptoms, function or health problems, and thus capture a Health Perceptions construct, specifically the “satisfaction with health” outcome. The *HPQ* is an important extension of single items, which covers six domains: prior health, current health, health outlook, resistance/susceptibility to illness, health worry/concern and sickness orientation. The developers of the *HPQ* contended that this subjective concept has as much to do with a person’s feelings and beliefs as their actual health status [73]. The *IPQ* is based on a model of the cognitive representation of illness measured by eight domains: Consequences, Timeline, Personal control, Treatment control, Identity, Concern, Understanding, Emotional response [74].

All three instruments contain a standard list of questions, asking about a person’s perception of their current self. All are status questions, although the *self-rated health status* item can also be asked as a transitional question. The *HPQ* can be scored as a profile, comprised of six sub-scores, or an overall index can be created from 22 of the 33 items. The *IPQ* can be reported as a profile (the responses to the nine questions) or a summative index.

#### Psychometric properties

The *self-rated health status* item is quick to administer. It consistently predicts long-term outcomes such as mortality. This suggests that it reflects health trajectory, and not merely current health status [27], which may make it less suitable for use over the short to medium term. A body of research has shown that general questions, self-rated health included, are answered less reliably than specific questions [75]. Single items are, by necessity, less precise than multiple items, and less responsive to change [76]. The *HPQ* has shown good reliability and validity. The high stability over time suggests that it may be more useful as a personality indicator than an outcome measure that is responsive to change [27]. The *IPQ* is most suitable for relatively ill populations [74]. The *IPQ* has shown good reliability and validity in populations with illness, but has generally been used as a variable which is associated with various outcome measures, rather than a measure in its own right [77]. Responsiveness to change in primary care has never been tested. One paper has shown the measure to be responsive to change in secondary care [78] and the authors suggest that the role of medical interventions in shifting illness perceptions is an important and under-researched area [77].

#### Summary

The three health perceptions instruments are among the least responsive in this review. The constructs captured by them are similar to the Health Perceptions construct which arose in the prior qualitative study but also has some important differences, in that the items capture more general perceptions about health, which are less likely to be shifted by intervention.

## Discussion

### Key findings

As far as we are aware, this is the first systematic review of its kind for generic PROMs for primary care. This review identified PROMs that are potentially suitable for measuring a wide-range of outcomes in any adult primary care patient, and twenty of these were reviewed in detail. The two key findings of the review were that: to date, there is no instrument which comprehensively covers the outcomes commonly sought in primary care; and there are different benefits both to PROMs which measure status at a point in time, and PROMs which measure change directly. These findings will inform researchers selecting or developing PROMs to test interventions in primary care, and should have a subsequent effect on policy and patient care, as the conclusions of clinical research studies which inform healthcare policy depend on the PROM selected as an endpoint. Confirming that a gap exists by reviewing existing PROMs is a necessary first step in any PROM development [5]. Our findings provided sufficient grounds for proceeding with development of a PROM for primary care; and, following completion of this review, we developed and tested the Primary Care Outcome Questionnaire and made it publicly available [79].

### Strengths and limitations

Strengths of this review include a reproducible search strategy, developed in collaboration with a librarian, a set of clear inclusion and exclusion criteria, and publication of the longlist of excluded PROMs for transparency.

The search strategy successfully identified the twelve papers used in the iterative process of developing and testing. However, all systematic reviews have the potential of omitting relevant articles, including unpublished material. Our exclusion criteria omitted PROMs which captured a narrow construct, such as pain, fatigue, or anxiety. This limited the scope of the review to generic measures, and meant that modular measures, such as PROMIS [80], were excluded. The electronic search was limited to the Medline database, and one of the filters relied on keywords assigned by authors. Some papers describing the development and validation of PROMs for primary care may not have been picked up by this strategy. However, although the use of keywords in a filter is not usually recommended in systematic reviews [81], it can be justified when the unit of analysis is the PROM, not the paper, because all PROMs alluded to in abstracts were followed up, which meant that even if the original development paper of a PROM was not identified by the search strategy, it could be identified through other means. Moreover, the search was supplemented by forward and backward reference searching, review of the Oxford PROMs Bibliography, and consultation with experts. Lastly, the decision on which PROMs to include and exclude at abstract screening stage, and any data extraction was carried out by a single researcher. Independent screening and extraction would have helped to identify and minimise error. We have attempted to be as transparent as possible by describing inclusion and exclusion criteria and by publishing a longlist of all potential PROMs identified in Additional file 2.

### Comparison with the literature

Systematic reviews of health status measurement instruments are often poorly conducted with many studies having a poorly described search strategy, using only a single database and failing to report whether data extraction was done by two independent reviewers [82].

We followed PRIMSA and COSMIN guidelines in this review, diverging from these only where there were clear reasons. For example, we did not use the existing search strategy for measurement properties published by the COSMIN group [83], because it was highly sensitive (therefore over-inclusive) and did not fit the purposes of this review. The approach we took had much in common with other systematic reviews of PROMs on related topics (such as empowerment [14, 15], integrative medicine [16], and patient experience) [17]. For example, in their review of patient experience measures, Hudon et al. similarly relied on keywords for one filter, and took a similar approach to mapping the constructs captured by the instruments reviewed onto their defined construct of “patient-centred care”, excluding instruments which measured only a narrow part of this construct [17].

This review identified various benefits and downsides to standardised, transitional and individualised PROMs respectively. Standardised PROMs are most successful in terms of their psychometric properties. Item wording and selection of a scale for standardised strongly affect interpretation. The review contained five PROMs with transitional questions. As anticipated [84], these were more responsive than the status measures. Only one individualised PROM, *MYMOP* was included in the review. As with other individualised PROMs [85–88] this is recommended for completion only through interview.

None of the instruments reviewed provided coverage of all outcome groups identified in the prior qualitative study. Of the Health Status questionnaires, the most extensively tested is the *SF-36*. This is also the most responsive of the standardised status instruments reviewed. Individualised and transitional questionnaires show greater responsiveness to change. Of the Health Empowerment instruments, the *PEI*, *PAM* and *heiQ* provide the best combination of responsiveness to change and psychometric testing. The Health Perceptions instruments reviewed are all less responsive to change, and may measure a form of health perception which is difficult to shift in primary care.

## Conclusions

This systematic review is the first of its kind to identify papers describing the development and validation of generic PROMs suitable for measuring outcomes from primary care. It identified that, to date, there is no instrument which comprehensively covers the outcomes primary care patients seeks and primary care clinicians seek to deliver, and thus provided grounds for proceeding with development of a PROM for primary care. It also provides a reusable search strategy for the identification of papers describing primary care PROMs. Finally, it presents information on a range of instruments which measure health status, health empowerment and health perceptions, and a critique of their strengths and limitations for use in primary care, which will be of benefit to other researchers in this field.

## Additional files

Additional file 1: Medline Search Strategy. Reproducible Medline search strategy, including the contents of all four filters. (DOCX 21 kb)

Additional file 2: Long-list of PROMs identified. Long-list of all PROMs identified through the first review of abstracts, as potentially meeting the inclusion criteria. The reasons for excluding these after the PROMs was reviewed are also documented. (DOCX 44 kb)

Additional file 3: Data extraction sheet column headings description. Description of the column headings and possible categories for the data extraction sheet, used to create Figs. 1–3. (DOCX 16 kb)

Additional file 4: List of instruments reviewed. Referenced list of the twenty instruments reviewed with a short description of each instrument. (DOCX 42 kb)

## Abbreviations

CAM-3: Three scales for Complementary and Alternative Medicine
CIMOS: Complementary and Integrative Medical Outcomes Scale
CMP: Change in Main Problem
EC-17: Effective Consumer Scale – 17 items
EQ-5D: European Quality of Life-5 Dimensions
heiQ: Health Education Impact Questionnaire
HPQ: Health Perceptions Questionnaire
ICECAP: ICEpop CAPability measure
IPQ: Illness Perceptions Questionnaire
MYMOP: Measure Yourself Medical Outcomes Profile
NICE: National Institute for Health and Care Excellence
ORIDL: Outcome in Relation to Impact on Daily Living
PAM: Patient Activation Measure
PEI: Patient Enablement Instrument
PE-LTCs: Patient Empowerment in Long-Term Conditions
PPQ: Patient Perception of Quality
PRISMA: Preferred Reporting Items for Systematic Reviews and Meta-Analyses
PROM: Patient-reported outcome measure
SF-12: Short Form 12
SF-36: Short Form 36
SRHS: Self-Rated Health Score
