# Supplementary material for: Identification, description and appraisal of generic PROMs for primary care: a systematic review
Source: BMC Fam Pract. 2018 Mar 15;19:41. doi: 10.1186/s12875-018-0722-9 (PMC5856382; doi:10.1186/s12875-018-0722-9)
Supplement: Supplementary file 1 — Medline Search Strategy. Reproducible Medline search strategy, including the contents of all four filters. (DOCX 21 kb) [file 12875_2018_722_MOESM1_ESM.docx]

Additional File 1: Structure Review Search Strategy

|  | PASC | Search Terms | Purpose |
| --- | --- | --- | --- |
| 1 | Population (Primary Care, minor or chronic conditions) | Primary Health Care/ or  Family Practice/ or  Physicians, Family/ or  General Practice/ or  Family Health/ or  nurse practitioners/ or  primary care nursing/ or  family nursing/ or  ((primary or family or community or patient-centred) adj (care or health$)).ti,ab,jw. or  ((general or family or nurs$) adj1 (practice$ or practitioner$)).ti,ab,jw. or  Ambulatory Care Facilities/ or  Ambulatory Care/ or  Ambulatory.ti,ab. or  Outpatient/ or  Outpatient$.ti,ab. or  Chronic Disease/ or  (chronic adj (illness or condition$ or disease)).ti,ab. or  self care/ or  self care.ti,ab. or  (minor adj (illness or condition$ or injur$)).ti,ab. or  Community Health Services/ or  Community Health Nursing/ or  Patient-Centered Care/ | This filter focussed the systematic review on PROMs developed for primary care, chronic conditions or minor illness. |
| 2 | **Aim** (Development or testing of PROMs) | ("Development and Validation").ti,ab. or  ("Development and Testing").ti,ab. or  (psychometric adj (properties or test$)).ti,ab. or  (reliab$ adj2 valid$).ti,ab. or  (Develop$ adj8 (measure or outcome measure or instrument or PROM or tool or patient reported or questionnaire or index or scale or profile)).ti,ab. Or  (Valid$ adj8 (measure or outcome measure or instrument or PROM or tool or patient reported or questionnaire or index or scale or profile)).ti,ab. or  (New adj4 (measure or outcome measure or instrument or PROM or tool or patient reported or questionnaire or index or scale or profile)).ti,ab. | This filter was designed to identify articles which described the *development or psychometric testing* of PROMs. This was necessary because the vast majority of papers which reference PROMs are trials or studies in which the PROM is an endpoint, not PROM development papers. |
| 3 | **Subject** (Outcome Assessment using questionnaires) | "Outcome and Process Assessment (Health Care)"/ or  Questionnaires/ or  "Outcome Assessment (Health Care)"/ or  Self Report/ or  Psychometrics/ or  Health Surveys/ or  Health Status Indicators/ or  Factor Analysis, Statistical/ | The aim filter identified references to development and testing of all “tools”, “measures” and “instruments”. However, these terms are used for interventions as well as questionnaires. I therefore included a filter using keywords to identify studies relating to outcome assessment using questionnaires. While this is not usually recommended in systematic reviews[1] it can be justified when the unit of analysis is the PROM, not the paper, because even if the search filters miss the initial paper which describes development of a PROM, that PROM will, in many cases, be described in another paper. Such an approach has been used successfully before.[2] |
| 4 | **Construct**  (Health Status, ability to impact health status, health perceptions) | "Patient Acceptance of Health Care"/ or  (Adhere$).ti,ab. or  Health Behavior/ or  (health$ adj (Behavio?r or life$ or living)).ti,ab. or  Health Knowledge, Attitudes, Practice/ or  patient Education as topic/ or  Self Care/ or  patient participation/ or  health services accessibility/ or  ((knowledge or understand$ or decision*) adj3 (health or condition or sickness or illness or self?manag$ or self?care or symptom$)).ti,ab. or  (patient-centred or empowerment or enablement or activation or self care or participation or access* or lifestyle).ti,ab. or  quality of health care/ or  (quality adj2 health$).ti,ab. or  ("quality of life" or $QoL or generic or patient generated or individuali#ed).ti,ab. Or  (function$ adj2 (health, physical, mental, social, status, assessment)).ti,ab or  (symptom$ or bothersome$).ti,ab. Or  Activities of Daily Living/ or  Health Status/ or  ((resilien$ or resist$ or susceptib$) adj2 (sickness or illness)).ti,ab. Or  Attitude to Health/ or  Patient Satisfaction/ or  ((Patient$ or health) adj2 (satisf$ or experience or confiden$)).ti,ab. or  Satisfaction.ti,ab. or  ((Perception$ or judgement$ or worry or concern or outlook) adj2 (Health or sickness or illness)).ti,ab. or  Sick role/ or  Self concept/ or  ("sick role").ti,ab. Or  ("self concept").ti,ab. or  Patient Safety/ or  ((safe$) adj2 (patient$ or care or health?care)).ti,ab. | The construct in question is multi-faceted and very wide, so this filter was very sensitive at the expense of precision, and included text words and subject headings relevant to the four constructs being measured. |

1. Higgins, J. and S. Green, *Cochrane Handbook for Systematic Reviews of Interventions Version 5.1.0* The Cochrane Collaboration, Editor. updated March 2011.

2. Hudon, C., et al., *Measuring patients' perceptions of patient-centered care: a systematic review of tools for family medicine.* Annals of Family Medicine, 2011. **9**(2): p. 155-64.
