# Supplementary material for: Identification, description and appraisal of generic PROMs for primary care: a systematic review
Source: BMC Fam Pract. 2018 Mar 15;19:41. doi: 10.1186/s12875-018-0722-9 (PMC5856382; doi:10.1186/s12875-018-0722-9)
Supplement: Supplementary file 2 — Long-list of PROMs identified. Long-list of all PROMs identified through the first review of abstracts, as potentially meeting the inclusion criteria. The reasons for excluding these after the PROMs was reviewed are also documented. (DOCX 44 kb) [file 12875_2018_722_MOESM2_ESM.docx]

**Additional File 2: Longlist of excluded instruments**

The 301 instruments which were longlisted but, on further review, excluded from the shortlist for the Structured Review are listed below, with reasons for exclusion.

| **#** | **Name of Instrument** | **Reason for Exclusion** | **Detail** |
| --- | --- | --- | --- |
| 1 | Activities health assessment | Intervention-specific | For occupational health. |
| 2 | Assessment of communication and Interaction Skills (ACIS) | Intervention-specific | For occupational health. |
| 3 | Assessment of Motor and Processing Skills (AMPS) | Intervention-specific | For occupational health. |
| 4 | Canadian Occupational Performance Measure | Intervention-specific | For occupational health. |
| 5 | Occupational Questionnaire | Intervention-specific | For occupational health. |
| 6 | 15-Dimensional Health-related Quality of Life measure | Population-specific | For adolescents. |
| 7 | 16-D | Population-specific | For adolescents. |
| 8 | Adult Primary Care Questionnaire | Construct | Measures patient experience. |
| 9 | Ambulatory Care Experiences Survey | Construct | Measures patient experience. |
| 10 | AQoL-4D and AQoL-6D | Construct | Measures general quality of life. |
| 11 | Arizona Integrative Outcomes Scale | Construct | Measures general quality of life. |
| 12 | ASCOT | Intervention-specific | For social care. Also measures general quality of life. |
| 13 | Assessment of Life Habits | Population-specific | For the elderly. Measures facilitation, function, QoL. |
| 14 | Assessment of Quality of Life Instrument | Construct | Measures general quality of life. |
| 15 | Barthel Index | Population-specific | Measures function in the elderly. |
| 16 | Beck Depression Inventory | Construct narrow | For depression. |
| 17 | Behaviour and Symptom Identification Scale (BASIS-32) | Construct narrow | For mental health. |
| 18 | Berg Balance Scale | Construct narrow | Measures balance in elderly. |
| 19 | BORG CR10 | Construct narrow | Measures breathlessness. |
| 20 | Brief Carroll Scale | Construct narrow | Measures depression. |
| 21 | Brief Symptom Inventory | Construct narrow | Measures depression. |
| 22 | Campbell Index of Well-being | Construct | Measures well-being. |
| 23 | CARE (Comprehensive Assessment and Referral Evaluation) | Population-specific | For the elderly. |
| 24 | Carver and Sheerer Self-Efficacy Scale | Construct | Measures self-efficacy. (not particularly health-related) |
| 25 | CAT-Health | Not available in English | In Spanish. |
| 26 | Center for Epidemiologic Studies Depression Scale | Construct narrow | Measures depression. |
| 27 | CHOICE Health Experience Questionnaire | Construct | Measures patient experience. |
| 28 | Chronic Pain Acceptance Questionnaire (CPAQ) | Construct narrow | Measures pain. |
| 29 | Cleveland Global Quality of Life Scale | Construct | Measures general quality of life |
| 30 | Client Empowerment Scale | Construct | Measures empowerment as internal construct only. Borderline regarding inclusion. 44 items so long. Strong focus on empowerment as control with a bias towards people who want to be firmly in control of their own health, and activist for improvement for others in similar conditions. |
| 31 | Client Generated Index | Construct | Measures general quality of life. Also interviewer administered. |
| 32 | Clinical Global Impression | Not patient-reported | Clinician reported (for mental illness). |
| 33 | Clinical Global Impression-Improvement Scale | Not patient-reported | Clinician reported (for mental illness). |
| 34 | Clinical Outcomes in Routine Evaluation – Outcome Measure (CORE-OM) | Construct narrow | Mental Health. Sounds generic, but is actually for psychiatric outpatients, and the questions reflect this. |
| 35 | Components of Primary Care Index | Construct | Measures patient experience. |
| 36 | Comprehensive Quality of Life Scale | Construct | Measures general quality of life. |
| 37 | Spiritual Well-Being Questionnaire | Construct | Measures spiritual well-being. |
| 38 | Consultation Care Measure | Construct | Measures patient experience. |
| 39 | Consultation Satisfaction Scale | Construct | Measures patient experience. |
| 40 | Coping Orientation for Problem Experiences inventory | Construct | Individualised measure of coping. |
| 41 | Coping Strategies Inventory | Construct | Measures coping. |
| 42 | Coping Strategies Questionnaire | Construct | Measures coping. |
| 43 | Core Outcome Measure Index (COMI) | Construct narrow | Measures physical function only. |
| 44 | Demand Control Questionnaire | Construct | Measures demands people have in their place of work and how well they cope with them. |
| 45 | Disease Repercussion Profile | Disease-specific | For rheumatoid arthritis. |
| 46 | Dougados Functional Index | Disease-specific | Measures only function. Designed for AS. |
| 47 | DTSQ(C) | Disease-specific | For diabetes. |
| 48 | DTSQ(S) | Disease-specific | For diabetes. |
| 49 | Duke Activity Status Index | Construct narrow | Measures physical function only. |
| 50 | DUKE Health Profile | Construct | Measures HRQoL, but excluded because nearly half the items measure self-esteem or social health. |
| 51 | (Mini) Duke UNC Health Profile | Construct | Excluded on the same basis as the DUKE. (This is the 10 item version.) |
| 52 | DUKE UNC Functional Social Support Tool | Construct | Measures social support. |
| 53 | EASY-CARE | Population-specific | For the elderly. |
| 54 | Edmonton Symptom Assessment System | Population-specific | For the terminally ill. |
| 55 | EORTC QLQ-C30 | Disease-specific | For cancer. |
| 56 | ERDA Questionnaire (emotional and Rational disease acceptance) | Construct | Measures disease acceptance. |
| 57 | EUROHIS-QO | Construct | Measures general quality of life. Also interviewer administered. |
| 58 | EUROPEP | Construct | Measures patient experience. |
| 59 | Family Hardiness Index | Construct | Measures the “4 Cs” of family hardiness. |
| 60 | Family Satisfaction Scale / Index | Construct | Assesses the satisfaction of family members in regard to family cohesion, flexibility and communication. |
| 61 | Ferrans and Powers Quality of Life Index | Construct | Measures HRQoL, but contains many non-health related aspects. (Importance-performance example.) |
| 62 | Flanagan Quality of Life Scale | Construct | Measures quality of life for the chronically ill/ healthy populations. Looks at quality of life only, not specific to health. |
| 63 | Frenchay Activities Index | Disease-specific | For stroke. |
| 64 | Functional Activities Questionnaire | Population-specific | For elderly. |
| 65 | Functional Autonomy Measurement System (SMAF) | Population-specific | For the elderly. |
| 66 | Functional Independence Measure | Population-specific | Following Rehab (basic activities of daily living). |
| 67 | Functional Limitations Profile | Population-specific | For the elderly. |
| 68 | Functional Status Index (FSI) | Disease-specific | For arthritis. |
| 69 | Functional Status Questionnaire | Construct | Overlaps with SF-36, but also includes more. However the extra domains it included (social and role function) did not emerge from my qualitative work as something that most people thought primary care had very much of an influence on. Does not measure pain, or health perceptions, unlike the SF36 so have therefore excluded it. Has 34 questions - not 36. |
| 70 | General Health Questionnaire | Construct | Actually a psychological questionnaire, although designed for use in general settings. Focuses on ability to carry out “normal functions”. |
| 71 | General Practice Assessment Questionnaire (GPAQ) | Construct | Measures patient experience. |
| 72 | General Practice Assessment Survey | Construct | Measures patient experience. |
| 73 | general symptom distress scale (GSDS) | Construct narrow | Measures specific symptoms. |
| 74 | Geriatric Depression Scale | Population-specific | For the elderly. |
| 75 | Glasgow Benefits Inventory | Intervention-specific | For use post-surgery. |
| 76 | Glasgow Health Status Inventory | Intervention-specific | For use post-surgery. |
| 77 | Glasgow Outcome Scale | Not patient-reported | For caregivers to complete after stroke. |
| 78 | Global Level of Functioning Scale | Not patient-reported | Clinician rated scale on function loss resulting from mental factors. |
| 79 | Global Life Satisfaction Scale (GLSS) | Construct | Measures quality of life. |
| 80 | Global Rating of Change Scale | Construct | This is the single item on change in health which appears in some questionnaires - e.g. COOP charts. I included the status version. |
| 81 | Goal Attainment Scaling | Construct | Measures achievement of goals. |
| 82 | Goldman Specific Activity Scale | Disease-specific | For cardiovascular disease. |
| 83 | Goteborg Quality of Life Instrument | Construct | Measures quality of life. |
| 84 | GP Patient Survey (including EQ-5D) | Construct | Measures patient experience. |
| 85 | Health and Activity Limitation Index | Construct narrow | This is a utility measure. Only asks about limitations in activities - not symptoms (e.g. pain, anxiety or depression.) |
| 86 | Health Assessment Questionnaire (Stanford Health Assessment Questionnaire) | Population-specific | For the elderly, or populations suffering debilitating illness. |
| 87 | Health concerns questionnaire | Construct | Measures health concerns. |
| 88 | Health Options Scale | Construct | Measure of health work: how families work together to promote health. Has some of internal empowerment in it but focus is on the family, not the individual. |
| 89 | Health Quotient Questionnaire (for CAM) | Construct | Measure of healthy living. Too narrow for inclusion. Is focussed on self-care and diet and contains specific items like: "I watch my calorie intake." |
| 90 | Health Self-Determinism Index (HSDI) | Construct | Measures beliefs about who is the right person to take care of health - the individual or the health professional. |
| 91 | Health Status Outcome Dimensions instrument | Population-specific | For the elderly, in receipt of nursing care. |
| 92 | Health Status Questionnaire - 12 | version superseded | Same as SF-36. |
| 93 | Health Status Questionnaire 2.0 | version superseded | Same as SF-12. |
| 94 | Health Utilities Index | Construct | Measures health status attributes, including sensation, mobility, emotion, cognition, self-care, pain, fertility. Deliberately narrow focus on function - dexterity, cognition, vision, speech, ambulation etc. Author thought that this was what was important, as it removed subjectivity and preference. Does not, therefore, really cover the relevant constructs. |
| 95 | Heinrichs Quality of Life Scale | Disease-specific | For schizophrenia. |
| 96 | Hopkins symptom Checklist | Construct narrow | For anxiety and depression. |
| 97 | Hospital Anxiety and Depression Scale | Construct narrow | For anxiety and depression. |
| 98 | How I feel about myself well-being scale | Construct | Measure of quality of life / happiness / wellbeing. |
| 99 | ICECAP-A | Construct | Focus on overall quality of life. Function is covered by the questions about independence and achievement and progress. |
| 100 | Illness intrusiveness | Construct | Asks how an illness "intrudes" in various aspects of life. |
| 101 | Individualised Care Scale | Not patient-reported | Measures nurse perceptions of secondary care. |
| 102 | Individually Prioritised Problem Assessment | Intervention-specific | Measures outcomes of assistive technology |
| 103 | Instrumental Activities of Daily Living Scale | Population-specific | For the elderly. |
| 104 | Jalowiec Coping Scale | Construct | Measures patient characteristics (coping) which may affect health outcome. |
| 105 | Karnofsky Performance Status Scale | Population-specific | For the terminally ill. |
| 106 | Katz Instrumental Activities of Daily Living | Population-specific | For the elderly. |
| 107 | Klein-Bell elimination scale | Not patient-reported | Clinician-reported. |
| 108 | Kohlman Evaluation of Daily Living Skill | Not patient-reported | Not patient reported - interview and task based. For long term metal illness. |
| 109 | Lancashire Quality of Life Profile (LQLP) | Disease-specific | For schizophrenia. |
| 110 | Late Life Function and Disability Index | Population-specific | For the elderly. |
| 111 | Lawton-Brody Instrumental Activities of Daily Living Scale | Population-specific | For the elderly. |
| 112 | Lee Functional Status Index | Disease-specific | For rheumatoid arthritis. |
| 113 | Leeds Disability Questionnaire | Disease-specific | For ankylosing spondylitis. |
| 114 | Lehman Quality of Life Interview | Population-specific | For mental illness. |
| 115 | Life Satisfaction in the Elderly Scale | Population-specific | For the elderly. |
| 116 | Life Satisfaction Index | Construct | Measures a single QoL construct (life satisfaction). |
| 117 | Life Satisfaction Scale | Construct | Measures a single QoL construct (life satisfaction). |
| 118 | Linear Analogue Self-Assessment Scales | Disease-specific | For cancer. |
| 119 | Liverpool Quality of Life Battery | Disease-specific | For epilepsy. |
| 120 | London Handicap Scale | Population-specific | For chronic, multiple or progressive disease. |
| 121 | Lower Extremity Functional Scale (LEFS) | Intervention-specific | For post-surgery / rehab. |
| 122 | Lubben Social Network Scale | Construct | Designed to measure social isolation in the elderly. |
| 123 | Maastricht Utility Measurement Questionnaire | Not available in English | Dutch Translation of the McMaster Utility Measurement Questionnaire. |
| 124 | MacAdam and Smith Scale | Population-specific | For palliative patients. |
| 125 | MacArthur Health and Behaviour Questionnaire | Population-specific | For children. |
| 126 | Macatar patient preference disability tool | Disease-specific | For arthritis. |
| 127 | Major Symptom Score Utility Index (MSSUI) | Disease-specific | For acute rhinitis. |
| 128 | Makenzie Functional Status Index | Intervention-specific | For use after an operation or intervention. |
| 129 | Manchester Short Assessment of Quality of Life (MANSA) | Construct | Measures life satisfaction. |
| 130 | McGill Quality of Life Questionnaire | Population-specific | For life-threatening illness. |
| 131 | McMaster Health Index | Too long | Contains 59 questions. |
| 132 | McMaster Toronto Patient Function Preference Questionnaire | Disease-specific | For musculoskeletal problems. |
| 133 | McMaster Utility Measurement Questionnaire | Disease-specific | For rheumatoid arthritis. |
| 134 | MD Anderson Symptom Inventory | Disease-specific | For cancer. |
| 135 | Measure the Quality of the Environment | Construct | Measures environmental characteristics. |
| 136 | ***Medical Interview Satisfaction Scale*** | Construct | Measures patient experience. |
| 137 | Medication Appropriateness Index | Construct | Measures appropriateness of prescribing for elderly patients. |
| 138 | Milwaukee Evaluation of Daily Living | Not patient-reported | For long term metal illness. Not patient-reported. |
| 139 | Mishel Uncertainty in Illness Scale | Intervention-specific | Measures "uncertainty in illness", which includes concepts of health perceptions and understanding as well as confidence in health systems and health professionals. Leans towards people who are quite sick and developed for cancer. Refers to "when I get out of hospital" |
| 140 | Missoula-VITAS Quality of Life Index | Population-specific | For palliative care. |
| 141 | Modified Spitzer Quality of Life Index | Disease-specific | For cancer. |
| 142 | Modified Williamsom Functional Assessment | Construct | This is for primary care, and was compared to the Mini-DUHP in a study by Bowman. I have not managed to locate the questionnaire. |
| 143 | MOS-General Adherence Scale | Construct | Measures adherence. |
| 144 | Multidimensional Assessment Schedule (MPI-SVaMA) | Population-specific | For the elderly. |
| 145 | Multidimensional Health Profile | Too long | Measures psychosocial health and health coping. Screening instrument designed to screen out the "worried well" by identifying traits like health anxiety, health literacy and coping. It is extremely long. |
| 146 | Multi-level Assessment Instrument | Population-specific | For the elderly. |
| 147 | Mutuality Scale | Construct | Measures extent of positive relationship between caregiver and receiver. |
| 148 | National Health Interview Survey | Too long | CDC developed survey used to give national information about the health of the nation. |
| 149 | Neck Disability Index (NDI) | Disease-specific | For neck disability. |
| 150 | Niemi Quality of Life scale | Disease-specific | For stroke. |
| 151 | Nottingham Extended Activities of Daily Living | Population-specific | For the elderly or disabled. |
| 152 | Nottingham HP | Population-specific | For the elderly or disabled. |
| 153 | OARS Multidimensional Functional Assessment Questionnaire | Population-specific | For the elderly. |
| 154 | Oswestry Disability Index | Disease-specific | For lower back pain. |
| 155 | Outcome Measure for spiritual healing | Intervention-specific | For spiritual healing. |
| 156 | [Outcome Questionnaire - 10 (45)](http://www.oqmeasures.com/page.asp?PageId=77) | Construct | Measures symptom distress, interpersonal functioning and social role. Designed as a measure of mental health vital signs. |
| 157 | Padilla and Grant Quality of Life Index | Disease-specific | For cancer. |
| 158 | Pain and Impairment Relationship Scale | Construct narrow | For pain. |
| 159 | Positive and Negative Syndrome Scale (PANSS) | Disease-specific | For schizophrenia. |
| 160 | Global Assessment of Functioning (GAF) | Not patient-reported | Clinician scoring system for the severity of illness in psychiatry. |
| 161 | Parent's perception of Primary Care | Construct | Measures patient experience. |
| 162 | Patient Assessment of Chronic Illness Care (PACIC) | Construct | Measures patient experience. |
| 163 | Patient Empowerment Scale (1) | Disease-specific | For cancer. |
| 164 | Patient Empowerment Scale (2) | Construct | Measures patient experience (although it refers to empowerment, it measures the empowering/ disempowering actions of doctors and nurses in hospitals.) |
| 165 | Kim Alliance Scale | Construct | Only measures external empowerment aspects (alliance with clinician and patient) therefore construct too narrow. |
| 166 | Treatment related empowerment scale | Disease-specific | For advanced HIV. |
| 167 | Health Care Empowerment Inventory | Disease-specific | For advanced HIV. |
| 168 | Patient Expectations and Evaluation Questionnaire | Construct | Measures patient expectations in hospital. |
| 169 | Patient Generated Index | Construct | Measures QoL. Individualised, and done through interview |
| 170 | Patient Health Questionnaire | Construct | Screening tool for mental disorders. |
| 171 | Patient Perceived Involvement in Care Measure | Construct | Measures patient experience. |
| 172 | Patient reactions assessment | Construct | Measures patient experience. |
| 173 | Patient Reported Outcomes Measurement Information System (PROMIS) | Construct | Measures a number of specific constructs. |
| 174 | Patient Satisfaction Scale | Construct | Measures patient experience. |
| 175 | Patient Specific Functional Scale | Intervention-specific | For use after operation. |
| 176 | Patient Specific Index | Intervention-specific | For use after hip replacement. |
| 177 | Patient-perceived patient-centeredness scale (PPPCS) | Construct | Measures patient experience. |
| 178 | Patient-related Index | Disease-specific | For chronic pelvic pain. |
| 179 | PEPS - Patient Evaluated Problem Score | Disease-specific | For cancer. |
| 180 | Perceived Health Competence questionnaire | Construct | Measures self-efficacy. |
| 181 | Perceived Impact of Problem Profile | Population-specific | For a specific injury / problem. |
| 182 | Perceived Improvement Questionnaire (PIQ | Population-specific | For recovering drug addicts. |
| 183 | Performance and Satisfaction of Activities of Daily Living (PS-ADL) | Construct narrow | Measures function only. |
| 184 | Personal Functional Goals Interview Protocol | Construct | Measures goals achieved through participation in a particular programme. |
| 185 | Personal Impact Health Assessment Questionnaire | Construct | Measures function. Developed for musculoskeletal problems /rheumatoid arthritis. Designed to measure disability. Has an importance / performance scoring system. |
| 186 | Personal Well-being Index | Construct | Measures quality of life. |
| 187 | Physical Self-Maintenance Scale | Population-specific | For the elderly. |
| 188 | Positive Well-Being Scale | Construct | Measures anxiety and depression. This is based on positive responses to the positive items of the GHQ-30. |
| 189 | PREOS - PC | Construct | Measures patients’ experiences of safety, and perceptions of outcome, in primary care. |
| 190 | Primary Care Assessment Survey (PCAS) | Construct | Measures patient experience. |
| 191 | Primary Care Assessment Tool | Construct | Measures patient experience |
| 192 | Problem Elicitation Technique | Disease-specific | For ankylosing spondylitis. |
| 193 | Profile of Mood States | Construct | Measures mood - e.g. levels of anxiety. |
| 194 | Psychological Adjustment to Illness Scale | Too long | Contains 46 questions. Borderline inclusion, because as well as function, it measures levels of support, trust in physicians and current perceptions of health, confidence in future health. However, nearly all the items refer to "your illness". Also it very specific in some domains - e.g. there are 7 questions on sexual function. |
| 195 | Psychosomatic Symptom Checklist | Construct | Measure symptoms based on a checklist. |
| 196 | QLICD-HY | Disease-specific | For hypertension. |
| 197 | QoL-GAP | Construct | Measures quality of life in mental health patients. (Uses importance/performance scale). |
| 198 | Quality of Caregiving Measure (CarerQol) | Intervention-specific | For outcomes of informal care. |
| 199 | Quality of Life Assessment Schedule | Disease-specific | For epilepsy. |
| 200 | Quality of Life Enjoyment and Satisfaction Questionnaire | Construct | There are 2 versions. A long (93 questions) and a short (16 questions). Both focus on overall quality of life not health. |
| 201 | Quality of Life in Depression Scale | Population-specific | For depression. |
| 202 | Quality of Life Inventory | Construct | Measures quality of life. |
| 203 | Quality of Life Profile -Senior Version | Population-specific | Measures quality of life. For older people. (importance performance scale) |
| 204 | Quality of Life Systemic Inventory (QLSI) | Construct | Measures quality of life. |
| 205 | Quality of Life Tool | Construct | Measures quality of life for caregivers. |
| 206 | Quality of Life-GAP | Construct | Measures quality of life using a goal attainment approach. |
| 207 | Quality of Well-being Scale QWB-SA | Too long | Contains 78 items. Normally interviewer administered |
| 208 | Quebec User Evaluation of Satisfaction with Assistive Technology | Construct | Measures user satisfaction with assistive technology devices. |
| 209 | Questionnaire of Changes in Experience and Behaviour | Not available in English | In German. |
| 210 | Questions on Life Satisfaction | Not available in English | In German. |
| 211 | QuiLL (Quality of Life in Later Life) | Population-specific | For the elderly. |
| 212 | QUOTE | Construct | Measures quality of care from the perspective of the elderly. Is mainly an experience measure. (Importance performance scaling.) |
| 213 | Rankin Scale | Disease-specific | For stroke. |
| 214 | RAPID3 (routine assessment of patient index data) | Disease-specific | For rheumatoid arthritis. |
| 215 | Rappaport Disability Rating Scale | Not patient-reported | For the cognitively impaired. Not patient reported. |
| 216 | Rating Question Form | Intervention-specific | For after LVAD implant. |
| 217 | Reintegration to Daily Living | Intervention-specific | For rehab. |
| 218 | Repertory Grid | Construct | Measures quality of life. |
| 219 | Roland-Morris Disability Questionnaire | Construct narrow | Measures back pain. |
| 220 | Rosenberg Self-Esteem Scale | Construct | Measures self-esteem |
| 221 | Rosser Index of Disability and distress | Construct | Measures function only. Includes an index of disability and one of distress. Has a wide range from no disability to confined to bed. None of other constructs included. |
| 222 | Rotterdam Symptom Checklist (RSCL) | Disease-specific | For cancer. |
| 223 | Rotter's internal/external scale / Rotter's locus of control scale | Construct | 13 questions to see how much control you think you have over life. Not health related. |
| 224 | Satisfaction With Life Scale | Construct | Measures quality of life. |
| 225 | Satisfaction Profile (SAT-P) | Construct | Measures quality of life. |
| 226 | Schalock and Keith Quality of Life Questionnaire | Population-specific | For people with intellectual disabilities. |
| 227 | Schedule for the Evaluation of Individual Quality of Life | Construct | Measures quality of life (individualised and interview administered) |
| 228 | Self-Evaluation of Life Function Scale (SELF) | Population-specific | For the elderly. |
| 229 | Self-Reporting Questionnaire | Construct | Measures mental health. |
| 230 | Self-Anchoring Striving Scale | Construct | Measures life satisfaction. |
| 231 | Self-Assessment of Treatment | Intervention-specific | Measures a narrow construct (pain). Also questions based on treatment for neuropathic pain. Asks questions like "How do you assess your pain relief after treatment in this study?" |
| 232 | Self-Care Assessment Schedule | Population-specific | For the elderly / infirm. |
| 233 | Self-Care Behavior Questionnaire | Not available in English | In Chinese. |
| 234 | Sense of Coherence Scale (Antonovsky's) | Construct | Construct invented by Antonovsky. Also called "Salutogenesis": a personal disposition to be able to cope and stay healthy |
| 235 | Severity of Illness Index | Not patient-reported | Clinician-reported. |
| 236 | Severity of the Main Complaint | Construct narrow | Measures symptoms. |
| 237 | Shona Symptoms Questionnaire | Not available in English | In Shona (Zimbabwean language) |
| 238 | Sickness Impact Profile | Population-specific | For the elderly / infirm. |
| 239 | Side Effects and Life Satisfaction Inventory (SEALS) | Disease-specific | For epilepsy. |
| 240 | SmithKline-Beecham Quality of Life Index (SBQOL); | Construct | Measures QoL: self now, ideal self, sick self. Based on the repertory grid technique. |
| 241 | Social Support Appraisals Scale | Construct | Measures subjective appraisal of social support. |
| 242 | Social Support Index | Construct | Measures subjective appraisal of social support. |
| 243 | Social Support List | Construct | Measures subjective appraisal of social support. |
| 244 | Social Support Scale | Construct | Measures subjective appraisal of social support. |
| 245 | Specific Activity Scale | Construct narrow | Measures physical function only. |
| 246 | Spiritual Health Inventory | Construct | Measures spiritual health. Questions seem appropriate for end of life. |
| 247 | Spitzer Quality of Life Index | Population-specific | For people with serious illness. |
| 248 | St George's Respiratory Questionnaire | Disease-specific | For COPD. |
| 249 | State-Trait Anxiety Inventory | Construct | Measures anxiety. Separates state anxiety (how I am at the moment) from trait anxiety (my underlying nature). |
| 250 | Strauss and Carpenter outcome scales | Disease-specific | For psychosis. |
| 251 | Subjective Health Complaints Inventory | Construct | List of common symptoms that may be psychosomatic (e.g. leg pain, insomnia) |
| 252 | Subjective Quality of Life Profile (SQLP) | Construct | Measure degree of satisfaction with various domains of life, the degree of change anticipated and the importance attributed to these domains. |
| 253 | Subjective Well Being Scale | Construct | Measures general quality of life. |
| 254 | Symptom Assessment 45 | Construct narrow | Measures symptoms. |
| 255 | Symptom Checklist-90-Revised (SCL-90-R) | Construct narrow | Measures symptoms. |
| 256 | Symptom Control Assessment (SCA) | Disease-specific | For cancer. |
| 257 | symptom frequency, bothersomeness and impact scales | Construct narrow | Measures symptoms. |
| 258 | Symptom Assessment 45 | Construct narrow | Measures symptoms. |
| 259 | The Four-Dimensional Symptom Questionnaire | Construct narrow | Measures symptoms. |
| 260 | The Picker patient Experience | Population-specific | For patients discharged from hospital. |
| 261 | The Reassurance Questionnaire | Construct narrow | Measures trust. |
| 262 | The Self-Care Measure | Construct narrow | Measures self-care. |
| 263 | The Subjective Domains of Quality of Life Measure (SDQLM), | Construct | Measures general quality of life. |
| 264 | Therapy Impact Questionnaire (TIQ) | Disease-specific | For cancer. |
| 265 | Support Team Assessment Schedule (STAS) | Disease-specific | For cancer. |
| 266 | Sendera Quality of Life Index | Disease-specific | For cancer. |
| 267 | Hospice Quality of Life Index (HQLI) | Disease-specific | For cancer. |
| 268 | McGill Quality of Life Questionnaire (MQOL) | Disease-specific | For cancer. |
| 269 | McMaster Quality of Life Scale (MQLS) | Disease-specific | For cancer. |
| 270 | Life Evaluation Questionnaire (LEQ) | Disease-specific | For cancer. |
| 271 | Assessment of Quality of Life at the End of Life (AQEL) | Disease-specific | For cancer. |
| 272 | Missoula-VITAS Index (MVQOLI) | Disease-specific | For cancer. |
| 273 | Todai Health Index | Construct | Measure for spurious or psychosomatic illness - very long. |
| 274 | Toronto Functional Capacity Questionnaire | Construct narrow | Measures function only. |
| 275 | Valued Life Activities Scale | Construct narrow | Measures function only. |
| 276 | WAYS (Ways of Coping Questionnaire) | Construct | Measures coping behaviours in a community-residing well population |
| 277 | WeeFIM | Population-specific | For children. |
| 278 | Well-Being Questionnaire | Construct | Measures well-being only. Developed for diabetes, but has since been used in other populations. |
| 279 | Western Ontario and McMaster Universities Osteoarthritis Index | Disease-specific | For osteoarthritis. |
| 280 | WHO Disability Assessment Schedule | Construct narrow | Measures function only. |
| 281 | WHO-5 | Construct | Measures psychological wellbeing. |
| 282 | WHO-BREF | Construct | Apart from a question on availability and quality of healthcare, and a section on function it is much more a measure of QoL - measures social relations, physical environment etc. 2 out of 4 domains not relevant. |
| 283 | WHOQoL-100 | Too long | Contains 100 items. |
| 284 | Wisconsin Quality of Life Index | Population-specific | Measures quality of life in people with severe mental illness. |
| 285 | Zung Self-Rating Depression Scale | Construct | Measures depression. |
| 286 | Psychological General Well-Being Index | Construct | Measures general well-being |
| 287 | MOS-6A | version superseded | Same as SF-6. |
| 288 | Knowledge, Attitude, and Self-Efficacy Asthma Questionnaire (KASE-AQ) | Disease-specific | For asthma. |
| 289 | Alzheimer Disease-Related Quality of Life Scale | Disease-specific | For Alzheimer’s. |
| 290 | progressive deterioration scale | Disease-specific | For Alzheimer’s. |
| 291 | Arthritis Impact Measurement Scale | Disease-specific | For arthritis. |
| 292 | Psychosocial Adjustment to Illness Scale | Construct | Measures adjustment to illness (psychosocial). |
| 293 | Occupational Questionnaire | Intervention-specific | For occupational health. |
| 294 | Interest Checklist | Intervention-specific | For occupational health. |
| 295 | Role Checklist | Intervention-specific | For occupational health. |
| 296 | Work assessments | Intervention-specific | For occupational health. |
| 297 | Volitional Questionnaire | Intervention-specific | For occupational health. |
| 298 | Sensory Integration Inventory | Intervention-specific | For occupational health. |
| 299 | Short Profile | Intervention-specific | For occupational health. |
| 300 | Sensory Behavioural Profile | Intervention-specific | For occupational health. |
| 301 | Patients' and Doctors' Expectations Questionnaire | Not patient-reported | Clinician-reported. |
