# Supplementary material for: Identification, description and appraisal of generic PROMs for primary care: a systematic review
Source: BMC Fam Pract. 2018 Mar 15;19:41. doi: 10.1186/s12875-018-0722-9 (PMC5856382; doi:10.1186/s12875-018-0722-9)
Supplement: Supplementary file 3 — Data extraction sheet column headings description. Description of the column headings and possible categories for the data extraction sheet, used to create Figs. 1–3. (DOCX 16 kb) [file 12875_2018_722_MOESM3_ESM.docx]

Additional File 3: Column headings of the data extraction sheet

|  | **Heading** | **Options** | **Description** |
| --- | --- | --- | --- |
| **Measurement Properties Data** | Items | Integer | Total number of items in the PROM? |
|  | Scale | Status (S) | The PROM scale capture status at a particular point or period in time. |
|  |  | Transitional (T) | The PROM scale captures change over a period in time. |
|  | Recall period | Free text description | Period over which the respondent has to recall either status or change. |
|  | Adaptability | Standardised (S) | The PROM is comprised of a list of standardised questions which all respondents answer by giving numeric responses or marks on the scale. |
|  |  | Individualised (I) | The questions are not standardised: respondents can select, identify or weight domains. |
|  | Dimensionality | Profile (P) | A score is produced for each domain |
|  |  | Index (I) | A single score is produced |
|  |  | Utility (U) | A single score is produced based on health-state preferences, derived from econometric utility theory, which can be multiplied by time in the health state to generate a QALY.[3] |
| **Construct** | 1a. Symptoms - Pain | Block highlighted, shaded or blank | **Block highlighted**: The construct explicitly covered, using similar language to the heading.  **Shaded**: The construct is covered using more general language, and is therefore implicit, depending on the interpretation of the item  **Blank**: The construct is not capture in the questionnaire. |
|  | 1b. Symptoms - Anxiety/depression |  |  |
|  | 1c. Symptoms - Other symptoms |  |  |
|  | 1d. Symptoms - Side-effects |  |  |
|  | 2a. Effects - Ability to do normal activities |  |  |
|  | 2b. Effects - Ability to enjoy life |  |  |
|  | 2c. Effects - Impact of illness on others |  |  |
|  | 3. Understanding of illness/ condition/ problems |  |  |
|  | 4. Ability to self-care/staying healthy |  |  |
|  | 5. Having and adhering to a plan |  |  |
|  | 6. Confidence in seeking healthcare |  |  |
|  | 7. Access to support |  |  |
|  | 8. Health satisfaction |  |  |
|  | 9. Health concerns |  |  |
|  | 10. On track for the future |  |  |
| Level of Psychometric Testing | Extent of Psychometric Testing | Extensive | The instrument has undergone an extensive amount of psychometric testing, validated in various populations in multiple countries, and has been cited in more than 1000 papers published by multiple authors. |
|  |  | Moderate | The instrument has undergone a moderate amount of psychometric testing, validated by studies in which the original authors are not involved, and has been cited in more than 100 papers. |
|  |  | Low | The instrument has been tested for validity and reliability, but reports of this are limited to a few papers, mostly by the original authors. |
|  | Level of responsiveness | Unknown | The instrument has not been tested for responsiveness, or has failed to show responsiveness in testing. |
|  |  | Low | The instrument has shown some evidence of responsiveness, although this may not have been in primary care. |
|  |  | Medium | The instrument has shown repeated evidence for responsiveness, including in primary care. |
|  |  | High | The instrument has shown change in primary care studies where other leading PROMs have not. |
