# Supplementary material for: Identification, description and appraisal of generic PROMs for primary care: a systematic review
Source: BMC Fam Pract. 2018 Mar 15;19:41. doi: 10.1186/s12875-018-0722-9 (PMC5856382; doi:10.1186/s12875-018-0722-9)
Supplement: Supplementary file 4 — List of instruments reviewed. Referenced list of the twenty instruments reviewed with a short description of each instrument. (DOCX 42 kb) [file 12875_2018_722_MOESM4_ESM.docx]

***Additional File 4: List of twenty instruments reviewed***

1. ***Short Form 36 (SF-36)*:** Designed by RAND as a generic indicator for use in population studies, this contains 36 items on physical and emotional symptoms and function, and includes dichotomous and adjectival response scales.[1]
2. ***Short Form 12 (SF-12)*:** Twelve item version of the *SF-36*.[2]
3. ***European Quality of Life - 5 dimensions (EQ-5D)***: Measures HRQoL as defined by five domains: mobility, self-care, usual activities, pain/discomfort and anxiety/depression. There is also a VAS for “current health”. A score is derived either directly from the VAS, or by applying a preference-based weighting.[3]
4. ***COOP Charts*:** Nine pictorial charts designed as a simple health screening tool in primary care. Each consists of a title, a question, and a five-point scale, illustrated by five pictures.[4]
5. ***Change in Main Problem (CMP)***: An item on the change in the main presenting problem. This can take a number of forms, but is normally a single transitional item with a Likert response scale. A validated format for this item is “thinking about the main problem you consulted your doctor with, is this problem…” on a five or seven-point Likert scale from very much better – very much worse.[5] [6] [7]
6. ***Measure Yourself Medical Outcomes Profile (MYMOP)***: Designed for primary care, or complementary and alternative medicine to measure the outcomes that patient considers most important. This has two individualised items on symptoms and activities and one standardised on well-being, scored on a scale from 0 to 6.[8]
7. ***Patient Perception of Quality (PPQ)***: Designed to measure quality in general practice, using Donabedian’s framework of structure, process and outcome.[9] The full instrument has 22 items, and the outcomes domain includes five items on a transitional five-point Likert scale.[10]
8. ***HowRU***: Designed to provide an even shorter measure than the *EQ-5D* for use in clinical practice, this has four items on discomfort, distress, disability and dependence, with a four-point adjectival scale.[11]
9. **Outcome in Relation to Impact on Daily Living** (***ORIDL)***: Developed to measure patient's views of the outcome of their care by asking about change, and relating this to impact on daily life. Contains two items on “your main complaint” and “overall well-being” scored on a nine-point transitional response scale.[12]
10. **Complementary and Integrative Medical Outcomes Scale *(CIMOS)***: Designed for patients receiving complementary and alternative (*CAM*) therapies, this instrument consists of 35 items in seven domains (pain, fatigue, physical function, personal control, existential, and general quality of life) in the format of statements. Responses range from 0 to 4 on a Likert scale.[13]
11. **Patient Activation Measure – 13 items (PAM-13):** This thirteen item Guttman-scaled instrument is designed to measure where patients are on a four-stage process of activation: from disengaged to activated.[14]
12. **Patient Enablement Instrument (PEI):** Six items which measure “enablement” (a construct related to coping, understanding and self-care) resulting from a GP appointment that day.[15]
13. **Health Education Impact Questionnaire (heiQ):** Forty items which assess the impact of patient education programs across a broad range of chronic conditions.[16]
14. **Effective Consumer Scale – 17 items (EC-17)**: Designed for use in self-management interventions, this measures the skills and attributes of an “effective healthcare consumer”. [17]
15. **Patient Empowerment in Long-Term Conditions (PE-LTCs):** Forty-seven items designed to measure empowerment in long-term conditions [18]
16. **Barriers to self-management and quality-of-life outcome** (**Barriers):** Questionnaire designed to measure barriers experienced by people with multiple long-term conditions, and their management of these barriers.[19]
17. **Three scales for Complementary and Alternative Medicine** (**CAM-3)**: Designed to measure the outcome of complementary and alternative medicine in 22 items.[20]
18. **Self-Rated Health Score (SRHS)**: There are a number of options for single item indicators of self-rated health, covered in McDowell (pg. 581).[21] They all contain a single item, which asks about health in general, sometimes in relation to age or personal circumstances. The item is rated against an adjectival scale: e.g. “taking everything into consideration, how would you rate your health in general these days?” excellent, good, fair, poor or bad.
19. **Health Perceptions Questionnaire (HPQ)**: Designed to measure health perceptions, through 33 items. Responses are on a five-point Likert scale, from “definitely true” to “definitely false” with a “don’t know” mid-point.[22]
20. **Illness Perceptions Questionnaire (IPQ)**: Designed to measure illness perceptions: the extent to whether an illness is perceived to be threatening or benign. It contains nine questions, with responses on an eleven-point scale from 0 to 10. Scale endpoints have verbal descriptions which differ for each question: e.g. the question “how concerned are you about your illness” is scored from 0 (not at all concerned) to 10 (extremely concerned).[23]

**References**

1. Ware, J.E., Jr. and C.D. Sherbourne, *The MOS 36-item short-form health survey (SF-36). I. Conceptual framework and item selection.* Medical Care, 1992. **30**(6): p. 473-83.

2. Ware, J., Jr., M. Kosinski, and S.D. Keller, *A 12-Item Short-Form Health Survey: construction of scales and preliminary tests of reliability and validity.* Medical Care, 1996. **34**(3): p. 220-33.

3. Brooks, R., *EuroQol: the current state of play.* Health Policy, 1996. **37**(1): p. 53-72.

4. Nelson, E.C., et al., *The functional status of patients. How can it be measured in physicians' offices?* Medical Care, 1990. **28**(12): p. 1111-26.

5. Kamper, S.J., C.G. Maher, and G. Mackay, *Global Rating of Change Scales: A Review of Strengths and Weaknesses and Considerations for Design.* The Journal of Manual & Manipulative Therapy, 2009. **17**(3): p. 163-170.

6. Campbell, J.L., et al., *Telephone triage for management of same-day consultation requests in general practice (the ESTEEM trial): a cluster-randomised controlled trial and cost-consequence analysis.* Lancet, 2014. **384**(9957): p. 1859-68.

7. Salisbury, C., et al., *Effectiveness of PhysioDirect telephone assessment and advice services for patients with musculoskeletal problems: pragmatic randomised controlled trial.* British Medical Journal, 2013. **346**(jan29 3): p. f43-f43.

8. Paterson, C., *Measuring outcomes in primary care: a patient generated measure, MYMOP, compared with the SF-36 health survey.* British Medical Journal, 1996. **312**(7037): p. 1016-20.

9. Donabedian, A., *The quality of care. How can it be assessed?* Journal of the American Medical Association, 1988. **260**(12): p. 1743-8.

10. Haddad, S., et al., *Patient perception of quality following a visit to a doctor in a primary care unit.* Family Practice, 2000. **17**(1): p. 21-9.

11. Benson, T., et al., *Evaluation of a new short generic measure of health status: howRu.* Informatics in Primary Care, 2011. **18**: p. 89–101.

12. Reilly, D., et al., *Outcome related to impact on daily living: preliminary validation of the ORIDL instrument.* BMC Health Services Research, 2007. **7**: p. 139.

13. Eton, D.T., L.M. Temple, and K. Koffler, *Pilot validation of a self-report outcome measure of complementary and alternative medicine.* Explore: The Journal of Science & Healing, 2007. **3**(6): p. 592-9.

14. Hibbard, J.H., et al., *Development and testing of a short form of the patient activation measure.* Health Services Research, 2005. **40**(6 Pt 1): p. 1918-30.

15. Howie, J.G., et al., *A comparison of a Patient Enablement Instrument (PEI) against two established satisfaction scales as an outcome measure of primary care consultations.* Family Practice, 1998. **15**(2): p. 165-71.

16. Osborne, R.H., G.R. Elsworth, and K. Whitfield, *The Health Education Impact Questionnaire (heiQ): an outcomes and evaluation measure for patient education and self-management interventions for people with chronic conditions.* Patient Education & Counseling, 2007. **66**(2): p. 192-201.

17. Kristjansson, E., et al., *Development of the effective musculoskeletal consumer scale.* Journal of Rheumatology, 2007. **34**(6): p. 1392-400.

18. Small, N., et al., *Patient empowerment in long-term conditions: development and preliminary testing of a new measure.* BMC Health Services Research, 2013. **13**: p. 263.

19. Bayliss, E.A., J.L. Ellis, and J.F. Steiner, *Barriers to self-management and quality-of-life outcomes in seniors with multimorbidities.* Annals of Family Medicine, 2007. **5**(5): p. 395-402.

20. Bann, C.M., F.M. Sirois, and E.G. Walsh, *Provider support in complementary and alternative medicine: exploring the role of patient empowerment.* Journal of Alternative & Complementary Medicine, 2010. **16**(7): p. 745-52.

21. McDowell, I., *Measuring Health*. 2 ed. 2006, New York: Oxford University Press.

22. Ware, J., *Scales for Measuring General Health Perceptions.* Health Services Research, 1976. **11**: p. 396-415.

23. Broadbent, E., et al., *The brief illness perception questionnaire.* Journal of Psychosomatic Research, 2006. **60**(6): p. 631-7.
